# Comparative efficacy and safety of traditional Chinese patent medicine for the treatment of type 2 diabetes mellitus: A Bayesian network meta-analysis protocol

**DOI:** 10.1097/MD.0000000000022564

**Published:** 2020-10-02

**Authors:** Jie Li, Sen Zhao, Yanqin Huang, Chuancheng Li, Bing Li, Yunsheng Xu

**Affiliations:** a First College of Clinical Medicine, Shandong University of Traditional Chinese Medicine, Jinan, Shandong Province; b Department of Traditional Chinese Medicine Department, The General Hospital of the People's Liberation Army, Beijing; c Department of Endocrine, Affiliated Hospital of Shandong University of Traditional Chinese Medicine; d Department of Endocrine, The Second Affiliated Hospital of Shandong University of Traditional Chinese Medicine, Jinan, Shandong Province, China.

**Keywords:** Bayesian, network meta-analysis, protocol, traditional Chinese patent medicine, type 2 diabetes mellitus

## Abstract

**Background:**

At present, the prevalence of type 2 diabetes mellitus (T2DM) has become a major public health issue throughout the world, especially in developing countries. Notably, traditional Chinese patent medicines (TCPMs) are of great significance in the treatment of T2DM combined with conventional Western medicine therapy. However, there is a lack of comparison among all the current common TCPMs for treating T2DM. Therefore, this study intends to explore the efficacy and safety of different TCPMs against T2DM through the Bayesian network meta-analysis (NMA).

**Methods:**

We will conduct a comprehensive and systematic search for randomized controlled trials (RCTs) of TCPM for the treatment of T2DM in both Chinese and English databases published till August 2020. Two researchers will be responsible for screening eligible literature, extracting data, and assessing the risk of bias of included studies independently. Then, pairwise meta-analyses and Bayesian network meta-analyses will be conducted to assess all available evidence. In the end, data will be analyzed using STATA15.0 and WinBUGS1.4.3 software.

**Conclusion:**

This study will compare the efficacy and safety of different TCPMs against T2DM in detail. Our findings will provide a reliable evidence for selecting clinical treatment program and guideline development of T2DM.

## 1 Introduction

Type 2 diabetes mellitus (T2DM) is characterized by elevated blood glucose level that results from disturbances of insulin secretion and insulin action or both; it refers to a complex disorder impacted by both lifestyle and genetic factors.T2DM and its severe complications like nerve damages,^[1]^ kidney diseases,^[2]^ and cardiovascular diseases^[3]^ have noticeably imposed societys medical burden over the past few decades. According to epidemiological investigations, it is estimated that global numbers of adult T2DM cases will rise to 592 million by 2035.^[4]^ Consequently, the prevalence of the disease has become a major public health problem worldwide, especially in developing countries. In recent years, complementary and alternative medicine has gradually been accepted and widely applied for treating T2DM. Many traditional Chinese patent medicine (TCPMs) are used as adjuvant drugs combined with conventional Western medicine therapy in the treatment of T2DM, including Jinlida granule, Xiaoke pill, Shenqi Jiangtang granule, Jinqi Jiangtang Tablet, Tianmai Xiaoke tablet, etc.[^5^,^6^,^7^,^8^,^9^,^10^,^11^,^12^,^13^,^14^] Besides, relevant treatment guidelines also highlight the important role of TCPMs (e.g., Jinlida granule) against this disease.^[15]^ However, a direct comparison of the efficacy and safety of different TCPMs for the treatment of T2DM is lacked, which affects the optimal choice of the clinical treatment plan. As an extension of the traditional meta-analysis, network meta-analysis (NMA) is able to analyze the relative effectiveness of different interventions through indirect comparisons among common reference groups. The biggest advantage of NMA is that it can quantitatively compare different interventions for the same disease, followed by ranking according to the efficacy of a certain outcome index, thereby providing evidence support for clinical drug selection.[^16^,^17^] The Bayesian method is a mainstream statistical model for reticular meta-analysis because of its more accurate estimation and flexible modeling.^[18]^

This study is the first Bayesian NMA to compare the efficacy and safety of commonly used TCPMs for treating T2DM, which take fasting blood glucose (FBG), 2-hour postprandial blood glucose (2-hPG) during 75-g OGTT, glycosylated hemoglobinA1c (HbA1c) and adverse events as the main efficacy indicators. This work will provide more comprehensive and reliable evidence-based medical evidence for the use of TCPMs affect T2DM in the clinical guidelines and the selection of the medicare drug list.

## 2 Materials and methods

### 2.1 Study registration

This NMA has been registered on the International Platform of Registered Systematic Review and Meta-analysis Protocols (INPLASY). The registration number is: INPLASY202080125 (DOI 10.37766/inplasy2020.8.0125).

### 2.2 Inclusion criteria

#### 2.2.1 Type of research

RCTs published in Chinese or English language for the treatment of T2DM with TCPMs, without restriction on the use of blind methods.

#### 2.2.2 Types of patients

Patients who have been diagnosed with T2DM will follow the American Diabetes Guidelines.^[19]^ There are no restrictions on gender, age, course of the disease, TCM syndrome, and race. The case number in the treatment group and the control group are both ≥30.

#### 2.2.3 Interventions

T2DM patients in the control group are treated with conventional Western medicine therapy, while those in the experimental group are treated with TCPMs combined with conventional therapy. The use of TCPM is limited to oral administration, regardless of the course and dose.

#### 2.2.4 Outcomes

The primary outcomes are the FBG, 2-hPG during 75-g OGTT, HbA1c, and adverse events (e.g., gastrointestinal symptoms, rash, hypoglycemia). The secondary outcomes are as follows:

1. Body mass index (BMI);
2. Fasting insulin and 2-h postprandial insulin;
3. Homeostasis model assessment-insulin resistance (HOMA-IR);
4. Homeostasis model assessment-β (HOMA-β).

### 2.3 Exclusion criteria

Exclusion criteria are as follows:

1. Non-clinical research types such as animal experiments and review, secondary research, and repeated publications;
2. Intervention measures are studies on the combination of multiple TCPMs;
3. Data reports are incomplete and impossible to be acquired;
4. T2DM patients had other comorbidities.

### 2.4 Database and search strategy

We have systematically studied the skills and precautions of literature retrieval before literature retrieval and worked out the final retrieval strategy after several pre searches. We will search the following sources regardless of date, language, or publication status: PubMed, Embase, Cochrane Central Register of Controlled Trials (CENTRAL), Cochrane Library, Web of Science, China National Knowledge Infrastructure (CNKI), Wanfang Database. We will apply a combination of medical subject headings (MeSH) and free-text terms, combined with a database-specific search specification to implement a search strategy. The search start and end time is from the establishment of the database to August 2020. All RCTs of TCPMs for T2DM will be searched systematically in this study. Besides, the references included in the systematic review/meta-analysis will be tracked. At last, we will also search for ongoing trials registered on the World Health Organization's International Clinical Trials Registration Platform. The details of PubMeds search strategy are shown in Table 1.

**Table 1:**
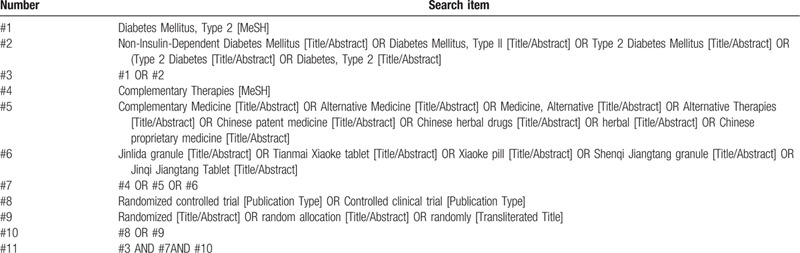
Detailed search strategy for Pubmed.

### 2.5 Data extraction

Literature management will be conducted using EndnoteX9 software. Two researchers (Jie Li and Sen Zhao) will be responsible for extracting data from eligible literature using Microsoft Excel independently. In case of disagreement, an independent third researchers (Yanqin Huang) will be consulted. When missing information that may affect the result of this NMA, we will try to contact the original study authors. The data of literature will be extracted are as follow:

1. General situation of the study, including title, authors, year of publication, journal, registration number to trial registries, country of conduct, and funded projects;
2. Basic information of patients, including sample size, age, gender, and duration of disease;
3. Intervention measures, including specific medication, dosage, and the course of treatment;
4. Outcomes, including primary and secondary outcomes, odds ratio (OR), mean difference (MD), confidence interval (CI).

### 2.6 Risk of bias assessment

Two researchers (Jie Li, Sen Zhao) will independently evaluate the methodological quality of the included studies, and resolve differences through discussion. The risk of bias will be assessed according to the Cochrane Handbook,^[20]^ which consisted of 6 items: Random sequence generation; Allocation hiding; Blinding of outcome evaluators; Blinding of patients and trial personnel; Incomplete result data; Selective reporting; Other biases (such as potential biases related to special research design, statement fraud, etc.). In the light of the relevant evaluation criteria, the included studies will be judged as “low risk of bias”, “high risk of bias” and “uncertain risk of bias”.

### 2.7 Assessment of heterogeneity

Because of the diversity of our research design, similar studies from different regions or countries will be all gathered for meta-analysis, which would inevitably result in differences. Heterogeneity will be solved using the following measures in this study:

#### 2.7.1 Subgroup analysis

If there is sufficient evidence, subgroup analysis will be conducted to explore the origin of heterogeneity in this study, including age, country, course of the disease.

#### 2.7.2 Sensitivity analysis

Sensitivity analysis will be performed by excluding each qualified literature. After excluding a study, if the heterogeneity changes, then this study may be the source of the heterogeneity. We will further analyze and explain the reason why the document became the origin of the heterogeneity. If the heterogeneity remains the same after excluding individual documents, then indicates that our results of this study are relatively robust.

### 2.8 Statistical analysis

#### 2.8.1 Statistical model selection

We will select the effect model on the basis of *I* ^2^ value and *P*-value for the heterogeneity test. When *I* ^2^ < 50% and *P* value >.1, it can be considered that there is no statistical heterogeneity in each study, so we use a fixed-effects model for meta-analysis. When *I* ^2^ ≥ 50% and *P* < .1, it can be confirmed that there is statistical heterogeneity among the studies, and the source of the heterogeneity needs to be analyzed. After excluding clinical heterogeneity factors (e.g., gender, age, the severity of the disease, and other factors), a random-effects model is used for meta-analysis. If there is clinical heterogeneity, subgroup analysis, and meta-regression analysis are needed to perform. Besides, if the source of the heterogeneity is unknown, meta-analysis is abandoned and descriptive analysis is applied.

#### 2.8.2 Pairwise meta-analysis

A pairwise meta-analysis will be conducted using STATA15.0 software. Dichotomous and continuous variables are expressed as OR and MD, respectively. 95%CI is calculated for each effect indicator. *I* ^2^ was calculated for reflecting the degree of heterogeneity among multiple studies.

#### 2.8.3 Network meta-analysis

STATA15.0 software will be used for NMA, and a random-effects model will be introduced to merge data and draw an evidence network. In the network, the thicker the arm indicates the larger the amount of basic data of the intervention, and the larger the circle area indicates the better the effectiveness of the intervention. The Bayesian NMA is mainly based on the Markov-chain-Monte-Carlo (MCMC), because it is more flexible and can solve the statistical processing in the complex evidence network. Moreover, it can use the posterior probability obtained to rank all intervention measures involved in the comparison and distinguish the good and bad order. Consequently, we will apply the MCMC in WinBUGS1.4.3 to perform Bayesian NMA of the random-effects model.^[21]^ When running the WinBUGS1.4.3 program, the number of iterations is set as 100,000, and the first 5,000 times are used for annealing to eliminate the influence of initial value. Besides, the Brooks-Gelman-Rubin statistical method is used to assess the convergence. At the same time, we will adjust the number of iterations and annealing time in the light of the specific situation, and calculate the 95% CI of the corresponding effect value. Moreover, this study will use the surface under the cumulative ranking curve (SUCRA) values to rank the intervention measures.^[22]^ The SUCRA value ranges from 0 to 1. The closer to 1, the better the possibility of intervention becoming the best intervention.

### 2.9 Assessment of inconsistency

When there are closed loops in NMA, its consistency is needed to be assessed. Thus, we will use the node splitting method to calculate the difference between the direct comparison evidence and the indirect comparison evidence and judge whether there is inconsistency through the *P* value.

### 2.10 Publication bias and evidence quality assessment

Potential publication bias in the situation with ten or more trials per comparison will be assessed by depicting Begg funnel plots in this study. In addition, the grading of recommendations assessment, development, and evaluation (GRADE) method is a commonly accepted approach to evaluate the quality of evidence and the strength of recommendations. It is well suitable for systematic reviews, health technology assessments, and clinical practice guidelines. Currently, GRADE is the most valuable tool for assessing the quality of evidence in the NMA. In the GRADE model, the quality of evidence is classified to high, medium, low, and very low, and the strength of recommendation was classified to strong and weak. Since RCT is the basis of the NMA, GRADE generally assess the NMA in the 5 aspects, that is, indirectness, inconsistency, imprecision, risk of bias, and publication bias.^[23]^

## 3 Discussion

Systemic evaluation/meta-analysis based on RCTs are significant sources of evidence for clinical practice, guideline formulation, and health-related decision making.^[24]^ However, traditional meta-analysis mainly focuses on the pair-wise comparison of intervention measures, which is unable to do multi-comparison analysis among various interventions. Therefore, a meta-analysis based on indirect comparison or a meta-analysis based on multiple intervention measures is needed, that is, network meta-analysis (NMA).[^16^,^17^,^18^] NMA is able to indirectly compare multiple interventions in relevant studies at 1 time, and rank the effects of interventions. It is conducive to comprehensively and fully analyze extracted data, thus enhancing the value of an individual RCT research.^[25]^ Notably, T2DM has become a major public health issue throughout the world, especially in developing countries. The pathogenesis of T2DM is not completely clear, and the main recognized mechanisms include insulin resistance, islet-β cell damage, and inflammatory response, etc. At present, conventional western medicines for the treatment of type 2 diabetes have some side effects, such as hypoglycemia, gastrointestinal discomfort, liver damage, etc. So, it is urgent to discover new and effective drugs with fewer side effects to cure T2DM. It should be noted that many TCPMs are applied in combination with conventional Western medicine therapy to the treatment of T2DM in clinical practice. TCPMs are made from Chinese herbal medicines and processed into various forms of Chinese medicine products, including pills, powders, granules, ointments, capsules, etc. TCPMs have several advantages, including ready-to-use, adaptable to urgent needs, convenient storage, easy to carry, eliminating the decoction process, peculiar smell, and adverse irritation compared with common Chinese medicine decoction. Pharmacological investigations indicated that TCPMs present beneficial effects on reducing body weight, enhancing insulin sensitivity, protecting β-cells, improving insulin secretion, correcting glucose and lipid metabolism disorders, and improving the microcirculation and the immune system.[^5^,^6^,^7^,^8^,^9^,^10^,^11^,^12^,^13^] For example, the Tianmai Xiaoke tablet which contains of Schisandra Chinensis, Ophiopogon Japonicus, Trichosanthis Radix and Chromium Picolinate is approved by the State Food and Drug Administration of China (state medical license number Z20049007) can decrease FBG and HbA1c levels in T2DM patients.^[26]^ In addition, some studies demonstrated that Tianmai Xiaoke tablets could down-regulate PTP1B and PCK2 by activating the insulin signaling pathway, thereby reducing the HbA1c level in diabetic rats.^[27]^ Moreover, the Jinlida granule was verified that it could significantly improve glycemic control, alleviate insulin resistance, and promote insulin secretion, with greater improvements in patients with a long disease course.^[28]^ A previous study demonstrated that Jinqijiangtang tablets could improve T2DM insulin resistance, regulating the gut microbiota and promoting the production of SCFAs. The mechanism was related to increasing the gut barrier function and reducing the host inflammatory reaction.^[29]^

To our knowledge, this study is the first report to introduce NMA based on existing RCTs, and evaluate and rank the advantages of various TCPMs against T2DM. The findings of this NMA will provide evidence support for clinical rational drug use, clinical program formulation, and medical insurance catalog screening. Notably, the quality of our NMA may be limited by the quality of the underlying data, such as publication biases in qualified literature. Thus, high-quality, multicenter studies are still necessary in the future to validate the efficacy and safety of TCPMs in the treatment of T2DM.

## Author contributions

**Conceptualization:** Jie Li, Yunsheng Xu.

**Data curation:** Jie Li, Sen Zhao.

**Funding acquisition:** Yanqin Huang, Yunsheng Xu.

**Methodology:** Bing Li.

**Project administration:** Yunsheng Xu.

**Software:** Chuancheng Li.

**Writing – original draft:** Jie Li.

**Writing – review & editing:** Jie Li.
